# Vegetation traits of pre-Alpine grasslands in southern Germany

**DOI:** 10.1038/s41597-020-00651-7

**Published:** 2020-09-28

**Authors:** Anne Schucknecht, Alexander Krämer, Sarah Asam, Abraham Mejia-Aguilar, Noelia Garcia-Franco, Max A. Schuchardt, Anke Jentsch, Ralf Kiese

**Affiliations:** 1 Karlsruhe Institute of Technology (KIT), Institute of Meteorology and Climate Research – Atmospheric Environmental Research (IMK-IFU), Garmisch-Partenkirchen, Germany; 2 WWL Umweltplanung und Geoinformatik GbR, Bad Krozingen, Germany; 3 German Aerospace Center (DLR), German Remote Sensing Data Center (DFD), Oberpfaffenhofen, Germany; 4 Eurac Research, Center for Sensing Solutions, Bolzano, Italy; 5 Technical University of Munich, TUM School of Life Sciences Weihenstephan, Chair of Soil Science, Freising, Germany; 6 University of Bayreuth, Bayreuth Center of Ecology and Environmental Research (Bayceer), Chair of Disturbance Ecology, Bayreuth, Germany

**Keywords:** Environmental sciences, Grassland ecology, Element cycles, Biogeography

## Abstract

The data set contains information on aboveground vegetation traits of > 100 georeferenced locations within ten temperate pre-Alpine grassland plots in southern Germany. The grasslands were sampled in April 2018 for the following traits: bulk canopy height; weight of fresh and dry biomass; dry weight percentage of the plant functional types (PFT) non-green vegetation, legumes, non-leguminous forbs, and graminoids; total green area index (GAI) and PFT-specific GAI; plant water content; plant carbon and nitrogen content (community values and PFT-specific values); as well as leaf mass per area (LMA) of PFT. In addition, a species specific inventory of the plots was conducted in June 2020 and provides plot-level information on grassland type and plant species composition. The data set was obtained within the framework of the SUSALPS project (“Sustainable use of alpine and pre-alpine grassland soils in a changing climate”; https://www.susalps.de/) to provide *in-situ* data for the calibration and validation of remote sensing based models to estimate grassland traits.

## Abstract (summary)

**Table:** 

Measurement(s)	canopy height • leaf mass per area • Green Area Index • total biomass yield • nitrogen content • carbon content • Coordinate • plant species specific cover
Technology Type(s)	rising plate meter • Planimeter • Analytical Balance • elemental analysis • GPS navigation system • visual observation method
Sample Characteristic - Organism	Viridiplantae
Sample Characteristic - Environment	temperate grassland biome • pre-Alpine grassland
Sample Characteristic - Location	Germany

Machine-accessible metadata file describing the reported data: 10.6084/m9.figshare.12738419

## Background & Summary

Grasslands cover about 40% of the total land area (excluding Antarctica and Greenland)^1^ and therefore represent an important ecosystem on the global scale. In Alpine and pre-Alpine landscapes, grasslands are a main element with a huge variability in their appearance ranging from intensively used grasslands in the lower regions to highly diverse seasonal mountain pastures and specialized natural ecosystems^2,3^. The (pre-)Alpine grassland ecosystems are mainly used for fodder production and thus provide significant economic value via meat and milk production^3^. Besides, these grassland ecosystems provide a multitude of other ecosystem services such as storage of nitrogen (N) and carbon (C), water purification and retention, as well as space for recreation and tourism^4–7^. Additionally, certain mountain grasslands represent some of the species-richest ecosystems in Europe^8–12^. In fact, these Alpine and pre-Alpine grasslands belong to the areas with the highest conservation priority in Europe (Council Directive 92/43/EEC of 21 May 1992).

Despite the economic value and the significant role of plants in the grassland C and N cycling, spatially explicit and reliable information on grassland management, biomass, and forage quality at the field and regional scale is rarely available. However, this information is needed to assess e.g. the regional C and N balances and to develop strategies for a sustainable management of these grasslands that secure the productivity and the various ecosystem functions. Remotely sensed data from unmanned aircraft systems (UAS) and satellites are one option to overcome this gap in the mapping and assessment of vegetation traits on a larger spatial scale^13^. In the last years, several studies made use of the potential of remotely sensed data to map grassland vegetation traits like above-ground biomass^14–21^ and quality parameters^22–27^. Geo-referenced *in-situ* data are of utmost importance for the development of remote sensing-based models for the estimation of grassland vegetation traits, for both calibration and validation purposes. However, the acquisition of *in-situ* data is time-consuming and labor-intensive. Therefore, the number of *in-situ* data sets with precise location information is limited in general and the accessibility is even lower.

**Fig. 1:**
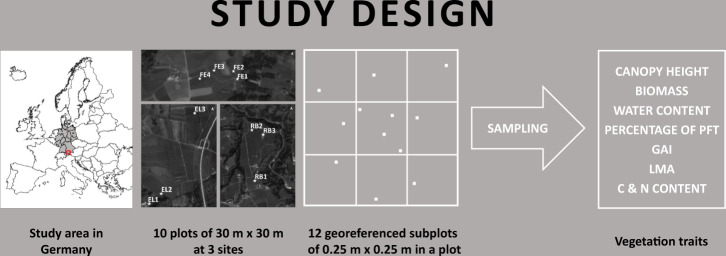
Schematic overview of the study design of the 2018 campaign. See Fig. 2 for the location of the three study sites.

The data set at hand intends to contribute to the body of freely available *in-situ* data on grassland vegetation traits and therefore to support the development of models for the spatially explicit estimation of grassland traits based on remote sensing data. We collected vegetation data of ten temperate pre-Alpine grassland plots in southern Germany in the TERENO Pre-Alpine Observatory (https://www.tereno.net/)^28,29^ via a sampling campaign in April 2018 (Fig. 1). Homogenous flat plots of 30 m × 30 m were selected and destructively sampled at nine to twelve 0.25 m × 0.25 m subplots. After the determination of the bulk canopy height, the vegetation was cut and the following parameters were determined in the laboratory for any of the sub-plots: weight of fresh biomass (biomass wm); weight of dry biomass (biomass dm); dry weight percentage of the plant functional types (PFT) non-green vegetation, legumes, graminoids, other forbs; total green area index (GAI) and PFT-specific GAI; C and N content of the PFT. Water content and mean (community) C and N contents were calculated from measured values. Additional samples around the subplots were taken to determine the leaf mass per area (LMA) of PFTs that occur in the plot. In total, the data set contains 120 observations with 32 measured or calculated parameters. In addition, a species inventory of all 10 plots was conducted in June 2020 to better characterize the plots in terms of grassland types and species composition.

The data set can be used for the calibration and validation of statistical models estimating grassland vegetation traits like biomass and plant N content based on remote sensing data. Besides, the data set could also serve as input or for validation of grassland plant growth routines of process-based models like the biogeochemical model LandscapeDNDC^30,31^, and in particular for regional upscaling procedures. Furthermore, the data can be used for the calibration of radiative transfer models like PROSAIL^32^.

## Methods

### Study area

**Fig. 2:**
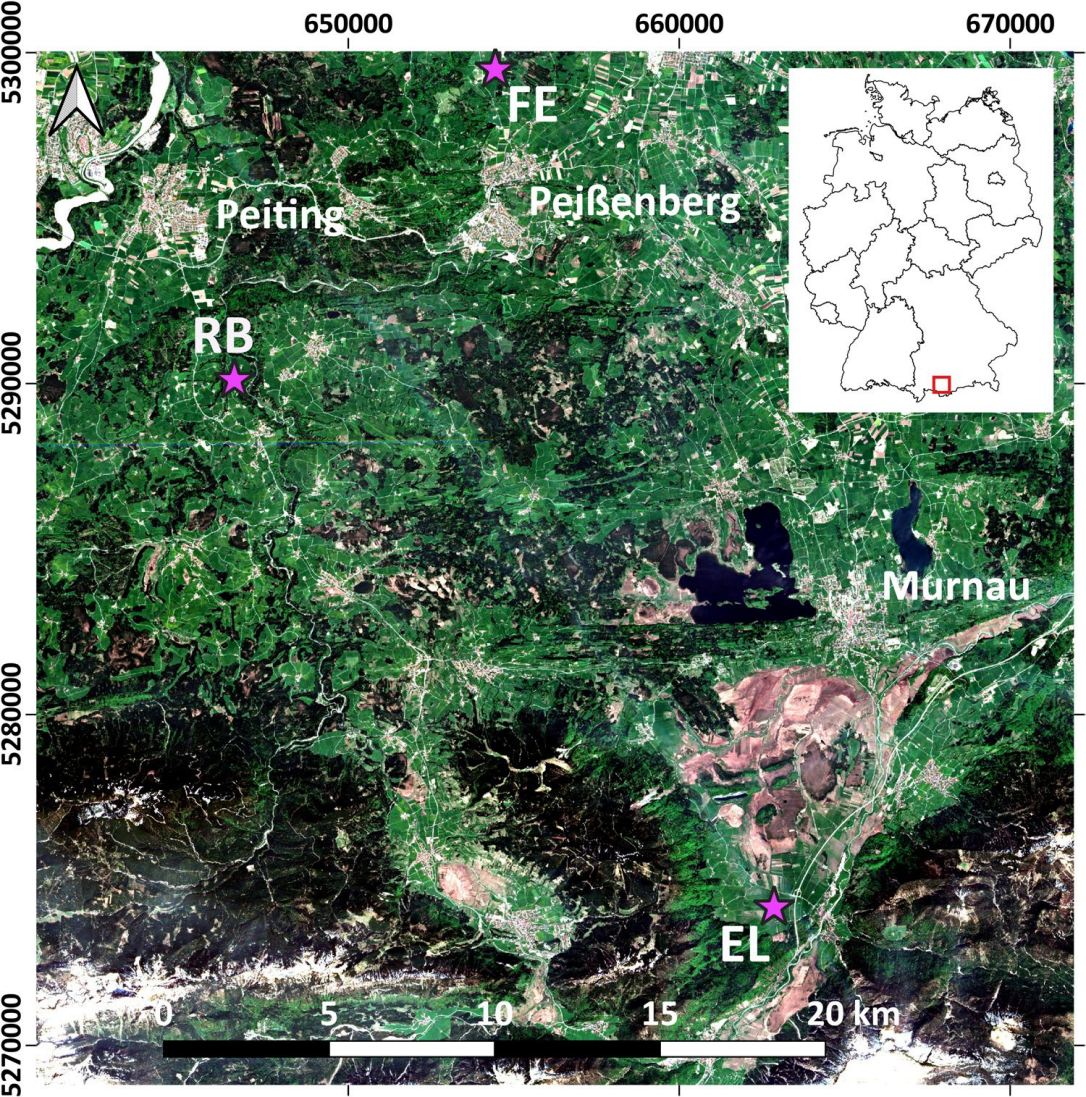
Location of the study sites (pink stars). Background: Sentinel-2b (27/04/2018), true colour composite (contains modified Copernicus Sentinel data [2018], processed by ESA). Used coordinate reference system: EPSG: 25832. EL = Eschenlohe, FE = Fendt, RB = Rottenbuch.

The study area is located in the TERENO Pre-Alpine Observatory^28,29^ in southern Bavaria, Germany (Fig. 2). The ten test plots are situated on three sites at different elevations: Fendt (FE), Rottenbuch (RB), and Eschenlohe (EL). Table 1 gives an overview about the main characteristics of these sites and the plots.

**Table 1:** Site and plot characteristics.

Site/Plot	Elevation [m a.s.l.]	MAP [mm]	MAT [°C]	Management	Grassland type	Species richness
Fendt (FE)	600	1008	8.6			
FE1				5 cuts, no pasture, 4x slurry	Arrhenatheretum elatioris	20
FE2				4 cuts, no pasture, 3x slurry	Arrhenatheretum elatioris	15
FE3				5 cuts, no pasture, 4x slurry	Arrhenatheretum elatioris	17
FE4				5 cuts, no pasture, 4x slurry	Arrhenatheretum elatioris	19
Rottenbuch (RB)	750	1159	8.0			
RB1				3–4 cuts, pasture, 4–5x slurry	Arrhenatheretum elatioris	30
RB2				5 cuts, no pasture, 5x slurry	Arrhenatheretum elatioris	25
RB3				1 cut, no pasture, no slurry	Caricion davallianae	44
Eschenlohe (EL)	630	1419	8.0			
EL1				1 cut, pasture, 2x slurry	Arrhenatheretum elatioris	17
EL2				4 cuts, no pasture, 4x slurry	Arrhenatheretum elatioris	23
EL3				3 cuts, no pasture, 2x slurry	Arrhenatheretum elatioris	27

Elevation values were obtained as the mean of all ground control point (GCP) elevation values of the corresponding site (rounded to the nearest ten). Mean annual climate parameters for the period 1981–2010 were retrieved from DWD Climate Data Center^35,36^. MAP = Mean annual precipitation height; MAT = Mean annual temperature. Grassland type and species richness were obtained by a species inventory in 2020 (see below).

Geologically, the plots in FE are located in the major structural unit of the molasse basin of the Bavarian Alpine foreland, while the other ones lie in the major structural unit of the Alps with the major tectonic units folded molasse (RB) and northern calcareous Alps (EL)^33^. Glacial erosion and Quaternary deposition processes influenced most of the molasse area. Therefore, alluvial structures and moraines largely effect soil parent material here^28^. The dominant soil types in the northern part of the study area are Cambisols, Luvisols, and Regosols, and in the southern part Rendzic Leptosols and Calcaric Cambisols. Gleysols and Histosols characterize areas along the course of rivers and areas of recent and paleo lakes^28^. According to the Köppen-Geiger climate classification the study area has a warm temperate climate without a dry season and warm summers (Cfb)^34^. Mean annual precipitation at the study sites varied between 1008 mm and 1419 mm^35^, and mean annual air temperature between 8.0 °C and 8.6 °C^36^ (see Table 1). The land cover around the study sites is characterized by a mix of pastures, natural grasslands, forests (needle-leaf, broad-leaf, mixed), discontinuous urban fabric, and in EL additionally peat bogs^37^. All ten plots are situated on managed grasslands, the dominant land use around the study sites. The management intensity of the plots range from very extensive management with only one cut and no fertilizer application per year to intensive management with five cuts and five slurry applications per year (Table 1).

### Sampling design

The field campaign with UAS flights and vegetation sampling took place on 24–25 April 2018 at ten different grassland plots (FE1, FE2, FE3, FE4, RB1, RB2, RB3, EL1, EL2, EL3). Plots were selected by i) visual characterisation of standing biomass to select plots that differ in management as well as soil nutrient and water status (based on talks with local farmers and corresponding field visits; no specific method was applied), and that fulfil other criteria such as ii) homogeneous, flat area, iii) accessibility (including permission by farmers), and iv) proximity of the plots to ideally cover several plots with one UAS flight.

When designing the sampling strategy, a perspective linkage of the sample data to Sentinel-2 images with a spatial resolution of 10 m × 10 m was taken into account. Therefore, we adapted the sampling strategy proposed by Baret *et al*.^38^ for the validation of medium spatial resolution land satellite products. The authors suggested relatively flat and homogeneous validation sites of 3 km × 3 km for validating data of sensors with a spatial resolution of up to 1 km × 1 km. Their validation sites were sampled at several so called elementary sampling units (ESUs, 20 m × 20 m). These ESUs were spread across the validation site using a division of the site in nine 1 km × 1 km squares (three to five ESUs per square) with a higher sampling density in the central square (five to seven ESUs)^38^.

In our study, we used 30 m × 30 m plots that were ideally sampled at 12 subplots (corresponding to the ESUs of Baret *et al*.^38^) of 0.25 m × 0.25 m (Fig. 1). We divided each plot in nine equally sized squares of 10 m × 10 m, in which we randomly placed one subplot. Following the suggestions of Baret *et al*.^38^, we sampled the central square with a higher density (i.e. with four subplots). Compared to Baret *et al*.^38^ we targeted a smaller number of subplots per plot (12 instead of 30 to 50) as our plot size is notably smaller than their plot size and hence it is easier to select a homogenous area. During the sampling campaign, we needed to reduce the number of subplots in the central square for EL1 (11 subplots were sampled) and EL2 (9 subplots) due to time constraints. In all other plots, we sampled 12 subplots.

### Preparations in the field

**Fig. 3:**
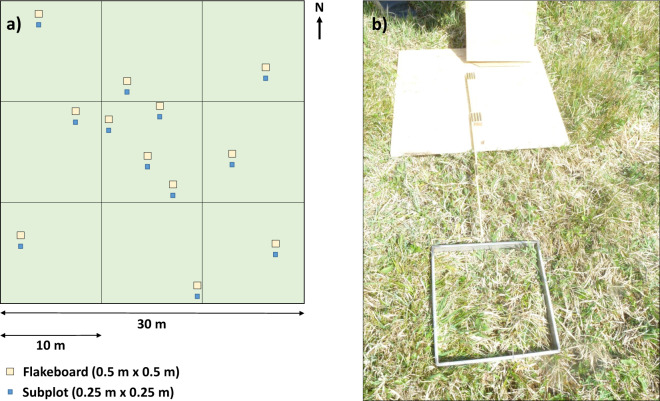
Sampling design. (**a**) Scheme of a sampling plot with subplots. The location of subplots within a 10 m × 10 m square was chosen randomly; (**b**) Location of a subplot with respect to the flakeboard.

Some arrangements needed to be done in the field prior to sampling to prepare accompanying UAS flights. The resulting images of these UAS flights were used among others for retrieving the exact location of the subplots. After the localisation of the plots in the field (aiming for a north-orientation of one plot site), bright 0.5 m × 0.5 m flakeboards were distributed in the plots at the approximate locations of the subplots (Fig. 3). These flakeboards were used to identify the location of the subplots in the orthophotos that were generated from images of the UAS.

Additionally, several ground control points (GCPs) were distributed in the overflight area of the UAS. The exact location of the GCPs’ centre was measured with a Global Navigation Satellite System (GNSS) receiver (Viva GNSS GS 10, Leica Geosystems AG, Switzerland) in static mode for 10 minutes. The data from the GNSS was reprocessed with Leica Geo Office 8.3 software (Leica Geosystems AG, Heerbrugg, Switzerland) utilising reference data from the satellite positioning service of the surveying administration of the federal states of Germany (SAPOS) for the real reference stations 0285-Garmisch, 0270-Bad Tölz, and 1271-Weilheim. The reference data were obtained via the SAPOS website of Bavaria (https://sapos.bayern.de/). The accuracy of the used GNSS in post-processing mode is 0.003 m in horizontal direction and 0.005 m in vertical direction^39^. The transformation of the corrected coordinates from ellipsoidal heights to physical (geoid-based) heights (height system: DHHN2016, EPSG 7837) was done with the online processing service “CRS-Transformation Bayern” from SAPOS (https://sapos.bayern.de/coord_tm.php). The transformation accuracy for this height transformation is 0.005 m^40^.

The UAS flights were conducted after the preparation of the respective field site, followed by the field measurements and vegetation sampling. A RGB camera (Sony Cyber-shot WX 220, Sony Corp., Minato, Japan) mounted on a fixed-wing UAS (eBee, senseFly, Cheseaux-sur-Lausanne, Switzerland) was used to acquire high-resolution images of the study sites. In total, four UAS-flights were necessary to cover all ten plots – one in FE, one in RB and two in EL (EL-N, EL-S).

### Acquisition of field measurements and field samples

The methods for acquiring *in-situ* data of canopy height, destructive vegetation sampling, and subsequent sample processing were adapted from the Integrated Carbon Observation System (ICOS) instructions for vegetation measurements in grasslands^41,42^.

#### Canopy height measurements and sampling for biomass and element content measurements

After the UAS flight, first the subplot area was identified (0.3 m south of the corresponding flakeboard, one site centred and parallel to the flakeboard, see Fig. 3b). Second, the bulk canopy height of the grassland canopy within the subplot was measured with a platemeter, which had the same area as the subplot for destructive sampling (0.25 m × 0.25 m) and was build according to the ICOS instructions^41^. The plate of the platemeter was constructed from acrylic glass and weighed 1680 g. Third, a metallic sampling frame (size: 0.25 m × 0.25 m × 0.03 m) was put on the subplot. After verifying that the sampling frame was not sliding on the vegetation, the vegetation within the sampling frame was clipped down to stubble height (0.03 m) with a manual grass cutter for later determination of biomass and element contents. Finally, the clipped vegetation was put in a labelled paper bag, then in an airtight plastic bag and afterwards in a cooling box until further processing in the laboratory.

#### Sampling for leaf mass per area determination

Additional samples were taken outside the subplots (within a radius of 2 m; one sample per subplot) to determine LMA. First, the area percentage of the PFTs legumes, other forbs and graminoids of the 30 m × 30 m plot was visually estimated (rough estimation based on field observations, no specific method applied). Then, the corresponding number of samples for each PFT was determined in relation to the number of subplots. That is, if the percentage of PFTs is e.g. 50% graminoids, 25% legumes, and 25% other forbs, and we have 12 subplots, there were six samples for graminoids, three for legumes and three for other forbs in this plot. At each subplot we took one LMA sample for just one specific PFT. One LMA sample consisted of fully expanded, undamaged leaves of the selected PFT originating from different individuals. The number of leaves per sample varied between one and seven depending on the leave size and the species composition of the PFT. However, the species composition could just be considered for dominant and easily distinguishable species, as the team was not specifically trained in grassland botany. Therefore, multiple species samples occurred mainly for forb samples. The clipped leaves were wrapped in humid paper. Then, the sample was put in a labelled plastic bag which was hermetically closed before it was placed in the cooling box.

### Sample processing in the laboratory

After transportation, the samples were stored in the fridge and/or in a cooling room at 4 °C until further processing. Sample processing in the lab started within one to nine days after the sampling date.

#### Samples for the determination of biomass, green area index, and element content

The samples were removed from the plastic bags and weighed. The weight of the fresh material was multiplied by 16 to determine the fresh weight per 1 m² (Biom wm). Afterwards, the sample material was sorted into the PFT non-green vegetation (NG; representing photosynthetically inactive structures), legumes (L), non-leguminous forbs (F), and graminoids (G). Then, the fresh weight of each PFT was determined. The sorting was done either on the full sample or on a representative smaller subsample in case there was a lot of sample material. For taking a subsample, the vegetation material was thoroughly mixed and then a handful of material was taken out. The remaining material was dried and further processed/analysed for C and N content like the PFT specific samples.

The total hemi-surface area of the green material (*GA*) needs to be obtained to determine the GAI of a sample. As we have flat vegetation structures, we used a planimeter (LI-3100A Area Meters, LI-COR, USA) to obtain the hemi-surface area. The hemi-surface area was measured separately for each PFT of a sample, but not for the non-green vegetation. The GAI of a certain PFT (*GAI*_*PFT*_) was then calculated by dividing the hemi-surface area of the PFT (*GA*_*PFT*_) by the area of the subplot (*A*; 0.25 m × 0.25 m) and in case a subsample was taken by multiplying with the ratio of the fresh weight of the sample (*Biomass wm*_*sample*_) to the subsample (*Biomass wm*_*subsample*_):

1 $$GA{I}_{PFT}=\frac{G{A}_{PFT}}{A}\frac{Biom\,w{m}_{sample}}{Biom\,w{m}_{subsample}}$$

The total GAI (*GAI*_*tot*_) of the sample was calculated by summing the GAI of each PFT:

2 $$GA{I}_{tot}=\sum GA{I}_{PFT}=GA{I}_{L}+GA{I}_{F}+GA{I}_{G}$$

After the planimeter measurements the samples were dried in an oven at 65 °C until constant weight was achieved. Then, the dry weight of each PFT of each sample was measured (*dm*_*PFT*_). The total weight of dry biomass of a sample (*Biomass dm*) was obtained by summing up the dry weights of all PFTs and if applicable the dry weight of the remaining material (*dm*_*rest*_) of a sample and then scaled to 1 m²:

3 $$Biomass\,dm=16\times \sum d{m}_{PFT}=16\times (d{m}_{NG}+d{m}_{L}+d{m}_{F}+d{m}_{G}+d{m}_{rest})$$

The percentage of each PFT (*P*_*PFT*_) with respect to the total dry biomass weight of the sorted material (either subsample or full sample) (Σdm_PFT_) was calculated as follows:

4 $${P}_{PFT}=\frac{d{m}_{PFT}}{\sum d{m}_{PFT}}\times 100=\frac{d{m}_{PFT}}{d{m}_{NG}+d{m}_{L}+d{m}_{F}+d{m}_{G}}\times 100$$

The plant water content (PWC) was calculated from the weight of the fresh (*Biom wm*) and dry biomass (*Biom dm*) as follows:

5 $$PWC=\frac{Biom\,wm-Biom\,dm}{Biom\,wm}\times 100$$

Finally, the dried vegetation material was ground in a ball mill for elemental analysis of C and N.

#### Samples for the determination of leaf mass per area

After returning from the field work, the leaves for the LMA determination were rehydrated until full turgescence. When the leaves were fully expanded, the last mature leaf from each tiller (in case tillers were sampled) were separated and the petiole was recut at the base of the leave blade. Then, the hemi-surface area of each sample was determined with the planimeter. Afterwards, the samples were dried in an oven at 65 °C until constant weight was achieved, before the dry weight of the LMA samples was measured. The LMA of a sample i was calculated by the ratio of the leaf dry weight (*W*_*i*_) to its fresh area (*A*_*i*_):

6 $$LM{A}_{i}=\frac{{W}_{i}}{{A}_{i}}$$

### Analysis of C and N content

The C and N content of the milled vegetation samples was determined using an elemental analyser (varioMax CUBE, Elementar Analysesysteme GmbH, Germany) operated in CNS (carbon, nitrogen, sulphur) mode with the plant method and a weighted sample of 17 mg at the laboratory of the Technical University Munich, Chair of Soil Science, in Freising (Germany). The detection limits of the instrument were 0.020 wt.% for C and 0.015 wt.% for N.

Due to the sorting of the sample in different PFTs, the C and N contents were obtained specific for each PFT. In cases where a subsample was taken for the sorting into PFT, C and N contents were additionally measured also for the mixed remaining sample material.

Mean concentrations of C and N for each subplot (plant community C and N) were calculated as follows:

7 $$\bar{E}={P}_{NG}\times {E}_{NG}+{P}_{L}\times {E}_{L}+{P}_{F}\times {E}_{F}+{P}_{G}\times {E}_{G}$$

where $\bar{E}$ is the mean element content (C or N), *P*_*PFT*_ is the percentage of the PFT (NG = non-green, L = legumes, F = non-leguminous forbs, G = graminoids), and *E*_*PFT*_ is the element content (C or N) in the PFT.

### Retrieving the coordinates of the subplot centres

The single images from each UAS flight were processed with the photogrammetric software PIX4D (Pix4Dmapper Pro, Pix4D S.A., Prilly, Switzerland) to obtain orthophotos. The GCPs in the orthophotos were used to georeference the orthophotos. The final spatial resolution of the orthophotos was 0.036 m (FE), 0.034 m (RB), 0.030 m (EL-N), and 0.043 m (EL-S).

Afterwards, the coordinates of the subplots centres were manually extracted from the high-resolution georeferenced orthophotos utilizing QGIS (Version 3.0.0-Girona)^43^. The bright flakeboards were visually localised in the images. Then the subplots centres were identified (0.425 m perpendicular away from the middle of the southern flakeboards site) and their coordinates extracted.

### Characterisation of grassland type and plant community composition on the plot-level

Vegetation relevés were carried out on 18–19 June 2020 for each of the ten 30 m × 30 m plots. Within each plot all vascular plant species were systematically determined and their cover was visually estimated to the nearest percentage as a proxy for abundance. Data is provided as percentage cover per plot. Plant species names were updated according to The Plant List, a working list of all known plant species, aiming to be comprehensive for all species of vascular plants, including flowering plants, conifers, ferns and their allies, and of bryophytes, including mosses and liverworts (theplantlist.org). The grassland type was classified sensu Oberdorfer (1977 and updated since)^44^. Please note that vegetation relevés from 2020 may slightly differ in relative coverage to actual species specific data from sampling in 2018. Nevertheless, most grassland species reach life spans of several decades and persist through time.

## Data Records

**Fig. 4:**
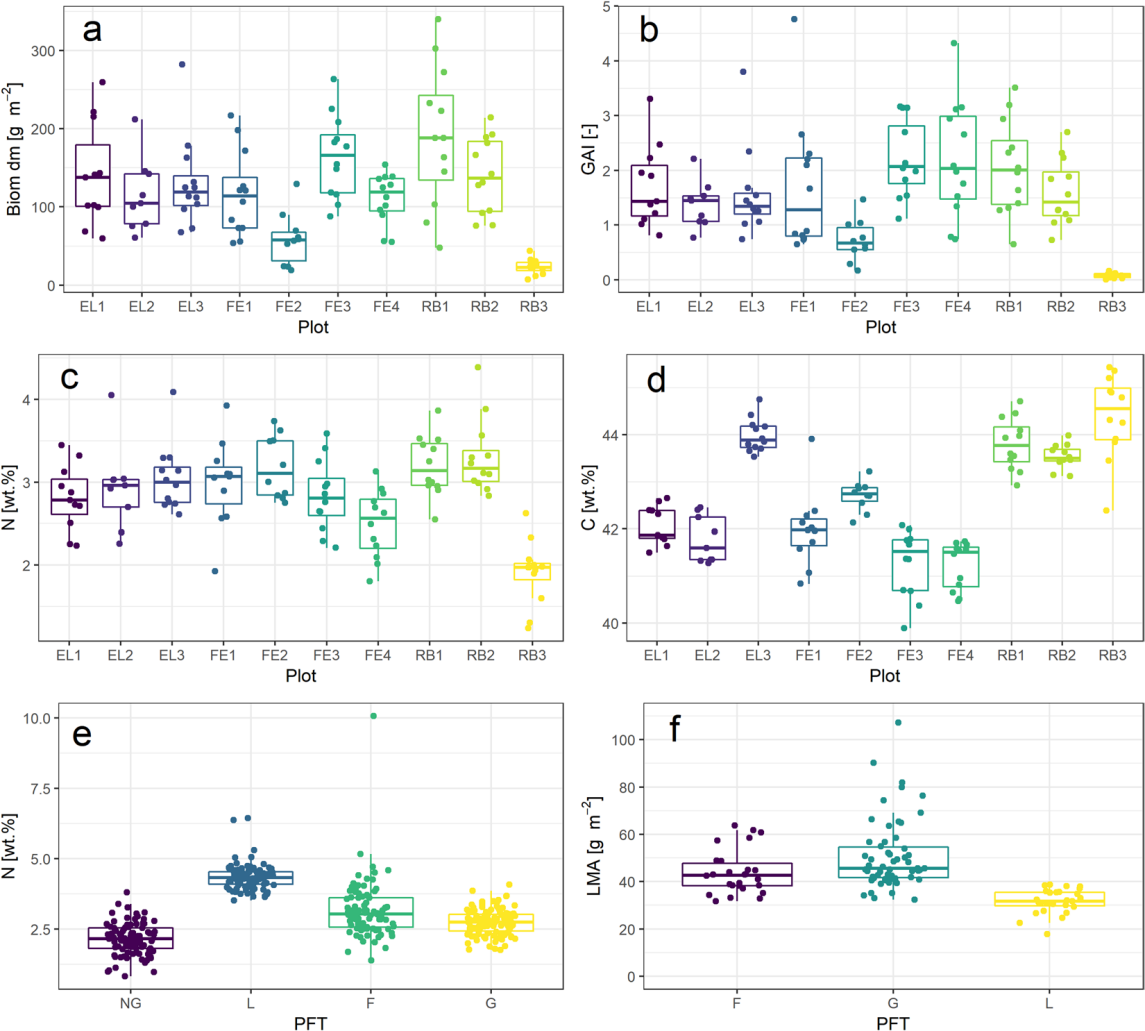
Selected grassland traits at different grassland plots (**a**–**d**) or for different PFTs (**e**,** f**). (**a**) weight of dry biomass per area (Biom dm); (**b**) total green area index (GAI_tot_); (**c**) plant community N content; d) plant community C content; (**e**) plant N content per PFT; (**f**) LMA values per PFT. F = non-leguminous forbs, G = graminoids, L = legumes, NG = non-green vegetation.

The data set was deposited at the PANGAEA repository as one data package^45^ with two data records consisting of i) the sub-plot specific data from the 2018 sampling campaign^46^ and ii) the plot specific species inventory from 2020^47^. Table 2 provides an overview about all sub-plot specific parameters of the data set from the 2018 campaign. The parameter short names were set by the PANGAEA team. The data set consists of 120 observations with 38 parameters. The first seven parameters characterize the observation (e.g. ID, location, sampling date) while the other 32 parameters correspond to measured or calculated traits. The Biom wm values and the derived PWC values are just indicative, as the samples of the different plots were taken at different times of the day and therefore might not be fully comparable. # indicates values that are not available due to various reasons (e.g., not sampled, sample lost, problems with measurement device). Examples for the value range and the distribution of grassland traits among the different plots are provided in Fig. 4 (a–d). Additionally, Fig. 4 (e, f) shows selected PFT-specific grassland traits.

**Table 2:** Overview and description of data set variables of the subplot-level data record based on the 2018 sampling campaign.

Parameter short name	Unit	Description	Example
Event	—	ID of the event	SUSALPS-RS2018_EL1-01
Subplot	—	ID of subplot	EL1-01
Latitude	°	y coordinate in EPSG 4326	47.5921016
Longitude	°	x coordinate in EPSG 4326	11.1586356
Date/Time	—	sampling date	2018-04-25
Site	—	site abbreviation	EL
Plot	—	plot abbreviation	EL1
Canopy h	cm	bulk canopy height; measured with a rising plate meter (RPM)	7.8
Leaf mass area	g m^−2^	leaf mass per area (LMA) for a certain PFT sample; calculated from dry weight of leafs (W) and fresh area of leafs (A) as *LMA = W/A*	46.2
PFT	—	plant functional type (PFT) of LMA sample	Graminoids
GAI legumes	—	green area index (GAI) of legumes; measured with planimeter	0.1
GAI graminoids	—	GAI of graminoids; measured with planimeter	1.1
GAI forbs	—	GAI of forbs; measured with planimeter	0.0
GAI tot	—	total GAI; calculated as sum of all GAI as *GAI tot = GAI legumes + GAI graminoids + GAI forbs*	1.1
Biom dm	g m^−2^	weight of dry biomass scaled to 1 m²	101.5
Biom wm	g m^−2^	weight of fresh (wet) biomass scaled to 1 m²	491.2
Non-green veg	%	percentage of non-green vegetation related to Biom dm	2
Legumes	%	percentage of legumes related to Biom dm	3
Forbs	%	percentage of forbs related to Biom dm	1
Graminoids	%	percentage of graminoids related to Biom dm	94
PWC	%	plant water content; calculated as PWC = (Biom wm - Biom dm)/Biom wm * 100	79.3
N cont non-green veg	%	nitrogen content of non-green vegetation as wt.%; measured with element analyser CNS	2.35
C cont non-green veg	%	carbon content of non-green vegetation as wt.%; measured with element analyser CNS	41.20
N cont legumes	%	nitrogen content of legumes as wt.%; measured with element analyser CNS	3.66
C cont legumes	%	carbon content of legumes as wt.%; measured with element analyser CNS	41.69
N cont forbs	%	nitrogen content of forbs as wt.%; measured with element analyser CNS	2.05
C cont forbs	%	carbon content of forbs as wt.%; measured with element analyser CNS	42.10
N cont graminoids	%	nitrogen content of graminoids as wt.%; measured with element analyser CNS	3.13
C cont graminoids	%	carbon content of graminoids as wt.%; measured with element analyser CNS	42.44
N org	%	nitrogen content of a mixed sub-sample (plant community N) as wt.%; measured with element analyser CNS	2.76
C org	%	carbon content of a mixed sub-sample (plant community C) as wt.%; measured with element analyser CNS	41.78
N org	%	mean nitrogen content of a sample (plant community N) as wt.%; calculated from percentage of each PFT and the corresponding N content: N org = Non-green veg * N cont non-green veg + Legumes * N cont legumes + Forbs * N cont forbs + Graminoids * N cont graminoids	3.12
C org	%	mean carbon content of sample (plant community C) as wt.%; calculated from percentage of each PFT and the corresponding C content: C org = Non-green veg * C cont non-green veg + Legumes * C cont legumes + Forbs * C cont forbs + Graminoids * C cont graminoids	42.39

The data record of the plot-specific species inventory contains the following variables: event ID, plot ID, site abbreviation, latitude, longitude, sampling date, grassland type (plant association), species richness, and species specific cover.

## Technical Validation

### C and N content

Plant C and N contents of one specific sample (this applies for PFT-specific and mixed samples) were measured multiple times and then averaged as it is relatively difficult to obtain homogenous plant samples due to different plant parts (vital and dead leafs, stems, pollen etc.) and different species in the sample. If the single measurements deviated notably from each other or showed suspicious high values, the measurement was repeated if there was still sample material available. Outliers (with more than 5% deviation from the mean of all single measurements of one specific sample) were not included in the final mean calculation of this sample. An internal standard was measured every 12 samples for quality control and was always in the acceptable range of less than 5% deviation from the expected values for C and N of the standard. Additionally, we assessed the value range of plant N content with literature values. Plant community N content in our data set (min = 1.2 wt.%, median = 2.9 wt%, max = 4.4 wt.%) is higher than in alpine grasslands of the Tibetan Plateau (min = 0.7 wt.%, median = 1.8 wt.%, max = 3.4%)^48^, but falls within the value range of plant community N content in temperate experimental grasslands in Germany (min = 1.0 wt.%, max = 4.6 wt.%)^49^.

### Leaf mass per area values

The plausibility of LMA values was checked by a comparison with literature values. LMA values ranged between 17.8 and 107.3 g m^−2^ (median = 42.0 g m^−2^). The value range between the 10^th^ and 90^th^ percentile of our data agrees well with the value range between the 10^th^ and 90^th^ percentile of grasslands as reported in the review of Poorter *et al*.^50^. The authors showed a median LMA of 59 g m^−2^ for grasslands^50^, which is slightly higher than the median LMA of our data set. As in the compiled grassland data set of Poorter *et al*.^50^ the median LMA of graminoids in our data set was higher than that of herbs, which are represented by legumes and non-leguminous forbs in our data (Fig. 4f).

### Potential sources of errors

Different potential sources of error can occur during sampling, sample processing, and sample analysis that could affect the values in our data set.

*Sampling sources of error* included vegetation losses during cutting (by wind or fall down) and that the vegetation was not exactly cut at 0.03 m within the subplot. These sampling error sources are likely to occur, but in general do not affect the biomass values too much. The effect of these errors are higher for subplots with low canopy height and therefore less biomass.

*Technical sources of error* were related to the planimeter measurements and sample handling. During planimeter measurements few sample material could be lost (by falling in the inside of the machine), leaves could partially overlap, and leaves could be measured several times when they stick on the conveyor belt. A change of water content during processing could affect planimeter measurement and the fresh weight of PFT subsamples. Errors related to the planimeter measurement are likely to occur, are of systematic nature and may lead in sum to a slight underestimation of GAI values. A few samples were affected by a slight mould development during the drying in the oven, which might be caused by a too tight placement and a missing ventilation in the oven. This is a rare error that could theoretically reduce the weight of the dry biomass through cell respiration of the fungi, but is not expected to have a measurable effect. For subtracting the dry bag weight from the weight of the dried sample within the bag, we often used a median value of several bags which could lead to a small error in the weight of the dry biomass. This error is higher for low sample weight to bag weight ratios. Another potential error is the incomplete homogenisation of the sample before taking a subsample (e.g. for PFT sorting, subsample taking for elemental analysis). An inhomogeneous subsample could lead to errors in the relative percentage of PFT and in element contents. The latter case was addressed by performing several elemental measurements on the same sample and then taking the average value.

## Usage Notes

The primary usage of this data set is the calibration and validation of remote sensing based models to estimate different grasslands traits. A study within the SUSALPS project (https://www.susalps.de/en/) currently utilizes the data set to analyses the potential of UAS-borne multispectral data to estimate Biom dm and plant community N content in pre-Alpine grasslands. Furthermore, the data set could be used as validation data for biogeochemical models like LandscapeDNDC^30,31^ or for the calibration of radiative transfer models like PROSAIL^32^.
